# Graphene Quantum Dots Decorated Gold-Polyaniline Nanowire for Impedimetric Detection of Carcinoembryonic Antigen

**DOI:** 10.1038/s41598-019-43740-3

**Published:** 2019-05-10

**Authors:** Akhilesh Babu Ganganboina, Ruey-An Doong

**Affiliations:** 1 Department of Biomedical Engineering and Environmental Sciences, National Tsing Hua University, 101, Section 2, Kuang Fu Road, Hsinchu, 30013 Taiwan; 2 Institute of Environmental Engineering, National Chiao Tung University, 1001 University Road, Hsinchu, 30010 Taiwan

**Keywords:** Biological techniques, Cancer

## Abstract

A label-free impedimetric immunosensor based on N, S-graphene quantum dots@Au-polyaniline (N, S-GQDs@Au-PANI) nanowires was fabricated for the quantitative detection of carcinoembryonic antigen (CEA). The N, S-GQDs and Au-PANI were synthesized by a simple hydrothermal pyrolysis and interfacial polymerization, respectively. Subsequently, 2–9 nm N, S-GQDs are successfully decorated onto 30–50 nm Au-PANI nanowires by Au-thiol linkage to serve as the bifunctional probe for amplifying the electrochemical activity as well as anchoring anti-CEA. The N, S-GQDs@Au-PANI nanowires are excellent conducting materials to accelerate the electron transfer, while the formation of CEA antibody-antigen bioconjugates after the addition of CEA significantly increase the charge transfer resistance, and subsequently provides a highly stable and label-free immunoassay platform for the impedimetric detection of CEA. The label-free immunosensor exhibits a wide linear range from 0.5 to 1000 ng mL^−1^ with a low detection limit of 0.01 ng mL^−1^. The N, S-GQDs@Au-PANI based immunosensor also shows high selectivity and stability over other cancer makers and amino acids. Moreover, this promising platform is successfully applied to the detection of CEA in human serum samples with excellent recovery of (96.0 ± 2.6)–(103 ± 3.8)%. These results clearly demonstrate a newly developed highly efficient and label-free impedimetric immunosensor for the detection of CEA using N, S-GQDs@Au-PANI nanowires as the biosensing probe, which can pave the gateway for the fabrication of high performance and robust impedimetric immunosensor to detect cancer makers in early stage of cancer diagnosis and therapy.

## Introduction

Carcinoembryonic antigen (CEA), a highly glycosylated protein with molecular weight of approximately 200 kDa, is one of the most widely used tumor markers for diagnosis, prognosis estimation and monitoring of malignant tumors such as pancreatic, colorectal, liver, breast and gastric cancers^1,2^. The level of CEA in the healthy human blood is in minimal level, while an elevated concentration of >5 ng mL^−1^ can be detected when cancer cells are formed. Immunoassay is one of the most widely accepted methods for tumor marker detection^3,4^. Various techniques such as electrochemical^5^, fluorescent^6^, electrochemiluminescent^7^, surface-enhanced Raman scattering^8^ and colorimetric^9^ methods have been adopted for the detection of CEA. Electrochemical sensor is one of the effective methods to detect CEA because of the advantages of high sensitivity, rapid response, easy to use and portability. Therefore, the development of sensitive, selective and label-free strategy of impedimetric detection of CEA can provide the essential information on antibody-antigen interaction for early tumor diagnosis and screening the disease recurrence.

The continuous development of nanotechnology offers a new horizon for electrochemical detection of cancer makers. Several nanomaterials such as quantum dots^10^, metal nanoparticles^11^ and conducting polymers^12^ have been used to amplify the detection signal in electrochemical immunosensors. Among the nanostructured materials used, noble metal nanoparticles such as gold nanoparticle (AuNP) has attracted increasing attention and become a promising material for ultrasensitive detection of chemicals and biomolecules^13,14^ have combined graphene and magnetic beads with enzyme-labelled antibody-AuNP bioconjugate for the electrochemical detection of CEA. However, AuNPs are quite easy to agglomerate in solution in the absence of support or immobilization matrix^13^. Nanostructured polyaniline (PANI) is a low-dimensional organic conductor with high surface area, which can exist in various protonated degrees ranging from the most reduced leucoemeraldine form to the half-oxidized emeraldine base form, and then to the fully oxidized pernigraniline form^15,16^. This property makes PANI a unique and interesting polymeric material. Moreover, PANI can serve as a stable support to offer a new route to interact with the immobilized nanoparticles for the enhanced electrochemical performance. Therefore, it gives a great advantage that the presence of uniformly distributed AuNP on the PANI surface can not only exhibit high electron transfer capability but also increase the amount of immobilized antibodies, thus significantly amplifying the signal source to enhance the sensitivity of the constructed electrochemical immunosensors.

As one of the promising materials in carbon family, graphene quantum dots (GQDs) have recently received extensive interest in biomedical applications due to their extraordinary electrical, mechanical and chemical properties, fast electron transfer kinetics and excellent electrochemical characteristics. The fabrication of GQD based nanocomposites as the novel biosensing probe has been reported to effectively detect biomolecules^17,18^. The structural defects of GQDs can be generated when heteroatoms are doped into the π-conjugated system, which can produce useful functionality for the enhancement of detection sensitivity toward targeted biomolecules and analytes^19–21^. A previous study has used N, S-codoped GQDs to detect Hg^2+^ ions and found that the chemically bonded N and S atoms could drastically enhance the fluorescence properties by altering the electronic characteristics as well as increasing the number of anchoring sites for the adsorption of metal ions^22,23^ Yang *et al*.^23^ have developed Pt-Pd/N-GQDs as the transducer to efficiently conjugate antibody as well as to amplify the electrochemical signal for CEA detection. Although GQDs are semiconductors, the excellent electrical properties, especially the high electron mobility and ballistic transport of charge carriers attributed to sp^2^-hybridized two-dimensional single-atom thick-layer structure, make them highly attractive on electrochemical sensing. However, the electrochemical detection of CEA by N, S-GQDs@Au-PANI based nanocomposites has rarely reported. The combination of N, S-GQDs with Au-PANI can form a novel nanocomposite, which possess the merits of organic and inorganic components, and may exhibit the unique properties that individual component does not have. In addition, the incorporation of N, S-GQDs onto Au-PANI nanocomposites gives a great impetus to fabricate a label-free immunosensor for ultra-sensitive detection of cancer makers, which can significantly enhance the electrochemical and sensing ability toward CEA detection.

**Figure 1:**
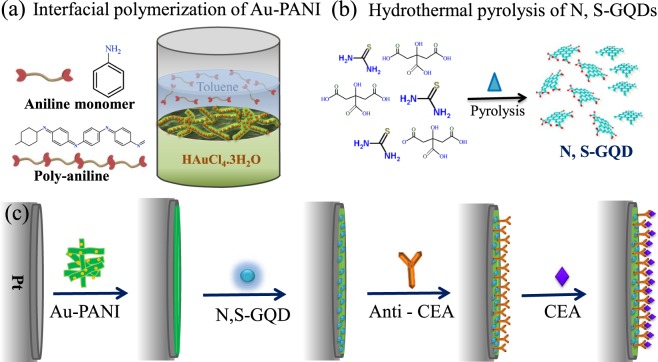
The schematic representation of (**a**) interfacial polymerization of Au-PANI, (**b**) hydrothermal pyrolysis of N, S-GQD and (**c**) fabrication of label-free CEA electrochemically impedimetric immunosensor.

Herein, we have developed an electrochemically impedimetric N, S-GQDs@Au-PANI immunosensor for the sensitive and selective detection of CEA. The detection principle of CEA is based on the change in impedance of N, S-GQDs@Au-PANI after the addition of CEA, which can inhibit the electron transfer after the formation of antibody–antigen bioconjugate on the N, S-GQDs@Au-PANI surface. As shown in Fig. 1a,b, Au-PANI and N, S-GQDs are fabricated by the interfacial polymerization and hydrothermal pyrolysis, respectively. After coating Au-PANI onto the Pt electrode, N, S-GQDs are then immobilized onto the Au-PANI surface by the Au-thiol interaction principle (Fig. 1c). N, S-GQDs are used as the bi-functional probe to amplify the electrochemical activity as well as to link antibodies. To the best of our knowledge, this is the first report on the immobilization of N, S-GQDs onto 1-D Au-PANI nanocomposites for impedimetric determination of CEA. The developed novel N, S-GQDs@Au-PANI nanocomposites show high sensitivity and good stability for quantitative determination of CEA in a linear range of 0.5–1000 ng mL^−1^ with limit of detection (LOD) of 0.01 ng mL^−1^, which exhibits a great potential in clinical and cancer diagnostic applications. The superior sensitivity of the developed impedimetric immunosensor is mainly attributed to the remarkable electro-conductivity of N, S-GQDs@Au-PANI, which can accelerate the electron transfer process between N, S-GQDs and electrode.

## Results

### Characterization of N, S-GQDs@Au-PANI nanowires

**Figure 2:**
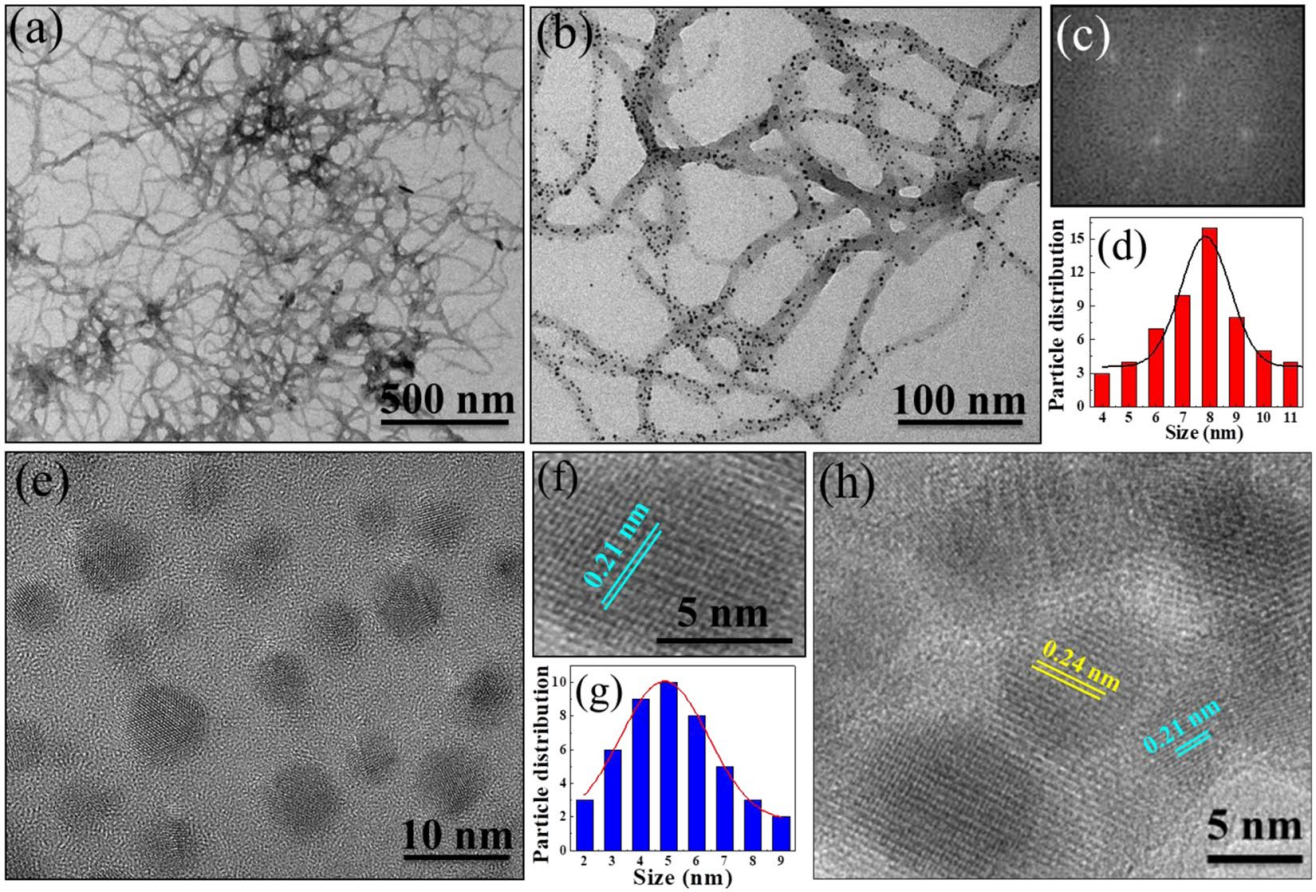
The (**a**,**b**) TEM images of Au-PANI nanowires, (**c**) fast fourier transform pattern and (**d**) particle size distribution of AuNP in Au-PANI nanocomposites, (**e**) TEM image, (**f**) HRTEM image and (**g**) particle size distribution of N, S-GQDs and (**h**) HRTEM image of N, S-GQDs@Au-PANI nanocomposites.

The TEM image is used to examine the morphology of as-prepared Au-PANI. As shown in Fig. 2a, the uniformly distributed Au-PANI nanowires are clearly observed. The regularly shaped Au-PANI nanowires are prepared by the interfacial polymerization method where aniline molecules in toluene are exposed to HAuCl_4_ in aqueous solution at the organic-aqueous interface to undergo the controlled polymerization. This prevents the branching and macroscopic precipitation of polymer, and subsequently generates PANI in nanoscopic scale. The HAuCl_4_ serves as the oxidizing reagent which can convert aniline to the polymeric conductive form of emeraldine salt, while Au^3+^ is reduced to its metallic form (AuNP) on the surface of PANI nanowires to form Au-PANI nanocomposites. The TEM image illustrated in Fig. 2b clearly show that the diameter of PANI is in the range of 30–50 nm where the AuNP are well dispersed within the polymeric matrix, which confirms the successful formation of nanocomposites. The fast Fourier transform pattern (Fig. 2c) shows the hexagonal lattice, depicting the (111) orientation of AuNP. From the histogram illustrated in Fig. 2d, the particle size of AuNP is in the range of 4–11 nm with mean diameter of 8 nm.

Figure 2e shows the TEM image of spherical N, S-GQDs with homogeneous distribution. The HRTEM is further used to identify the formation of N, S-GQD. As illustrated in Fig. 2f, the lattice fringe is calculated to be 0.21 nm, which corresponds to the (100) plane of as-prepared GQDs^24^. The particle size distribution of N, S-GQDs shown in Fig. 2g is in the range of 2–9 nm and the average lateral size is 4.8 ± 0.5 nm. Since the sulfur has high affinity toward AuNP, the N, S-GQDs can be intimately attached onto the Au-PANI nanocomposites via Au-thiol chemistry. Moreover, the surface morphology of N, S-GQDs@Au-PANI shown in Fig. 2h clearly indicates the homogeneous distribution of N, S-GQDs onto the Au-PANI nanowires. Two types of fringes at 0.21 and 0.24 nm are also deciphered, which match the characteristic lattice plane of N, S-GQDs and AuNP, respectively^25^.

Further characterization of N, S-GQDs@Au-PANI nanocomposites is performed by XRD. The XRD patterns reported in Fig. S1 (Supplementary Information) show the characteristic Au peaks at 2θ of 38.2°, 44.3°, 64.4°, 77.6°, and 81.8°, which correspond to the (111), (200), (220), (311) and (222) orientations of AuNP, respectively. In addition, a weak and broad peak centered at 25° 2θ is observed. This reflects the amorphous feature of PANI, which is similar to the previously reported Au-PANI nanocomposites^26^. Thermogravimetric analysis (TGA) of N, S-GQDs@Au-PANI along with the as-prepared Au-PANI is carried out in N_2_ to evaluate the loading of organic layer (Fig. S2, Supplementary Information). The total weight losses are found to be 42.8 and 55.2 wt% for as-prepared Au-PANI and N, S-GQDs@Au-PANI, respectively. The weight loss of Au-PANI in the temperature range of 30–200 °C corresponds to the removal of physically adsorbed water molecules and the decomposition of oligomer molecules in the PANI chains. The major degradation occurs in the temperature range of 200–580 °C, which can be attributed to the breakdown of PANI chains. An additional weight loss of 12.4% at 350–430 °C of N, S-GQDs@Au-PANI nanocomposites in comparison with bare Au-PANI can be assigned as the attached amounts of N, S-GQDs in nanocomposites. Moreover, the FTIR spectrum of N, S-GQDs shown in Fig. S3 (Supplementary Information) clearly indicates a broad peak at 3438 cm^−1^, which is the stretching vibration of the amino (N–H) and hydroxyl (O–H) groups on the surface. The graphitic layer of N, S-GQDs also can be confirmed by the C–H and C–C stretching at 1378 and 2076 cm^−1^, respectively, which is in good agreement with the reported results^21,22^.

**Figure 3:**
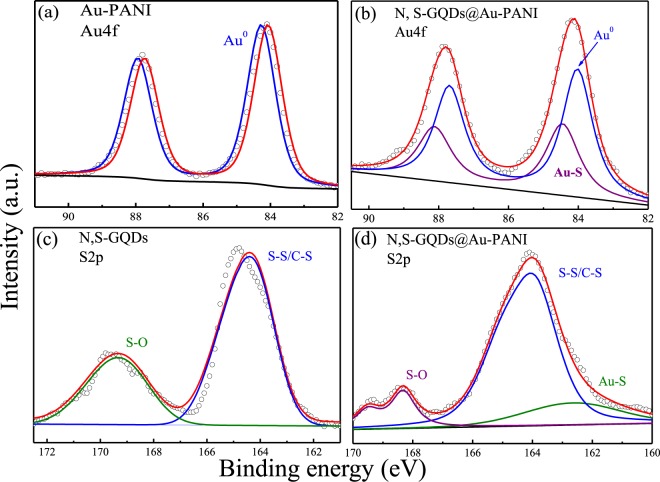
The deconvoluted XPS spectra of Au 4f of (**a**) Au-PANI and (**b**) N, S-GQDs@Au-PANI, and S 2p of (**c**) N, S-GQDs and (**d**) N, S-GQDs@Au-PANI.

XPS is carried out to identify the change in elemental environment before and after the loading of N, S-GQDs onto the Au-PANI nanowires. As shown in Fig. S4 (Supplementary Information), the survey spectrum of Au-PANI shows the standard peaks of C 1s (284 eV), O 1s (531 eV), N 1s (400 eV), Au 4d (335 and 354 eV) and Au 4f (85.8 and 83.9 eV). After the attachment with N, S-GQDs, two additional contributions at S 1s (228 eV) and S 2p (162 eV) are clearly observed, which are contributed from the S element in N, S-GQDs. The doped amounts of N and S in N, S-GQDs is estimated from the XPS full survey spectrum of N, S-GQDs in Fig. S4 (Supplementary Information) by using CasaXPS software. The doped amounts of N and S in N, S-GQDs are calculated to be 7.0 and 1.9 wt%, respectively. To explore the Au-thiol linkage between N, S-GQDs and Au-PANI in N, S-GQDs@Au-PANI nanocomposites, the Au 4f and S 2p peaks of N, S-GQDs@Au-PANI based nanomaterials are further deconvoluted. As shown in Fig. 3a, the deconvoluted Au 4f spectra of Au-PANI show the peak at 84.3 eV, which is mainly contributed from the elemental Au (Au^0^). After the linkage of N, S-GQDs, the Au^0^ peak in the deconvoluted Au 4f spectra of N, S-GQDs@Au-PANI shift to 84.0 eV (Fig. 3b), which is mainly attributed to the co-existence of electronegative sulfur element on AuNP^27^. The appearance of an additional peak at 84.4 eV confirms the formation of Au-S bond as N, S-GQDs can bind with Au-PANI. Similarly, the deconvolution of S 2p spectra of N, S-GQDs (Fig. 3c) also shows the formation of Au-S linkage. Two peaks at 169.3 and 164.3 eV can be assigned as the S-O and S-S bonds, respectively^22^. Moreover, the deconvoluted S 2p spectra of N, S-GQDs@Au-PANI exhibits an additional peak at 162.5 eV(Fig. 3d), which is also attributed to the formation of Au-S bond^28^ between N, S-GQDs and Au-PANI. These results clearly indicate the successful formation of N, S-GQDs@Au-PANI nanocomposites.

### Electrochemical characterization

**Figure 4:**
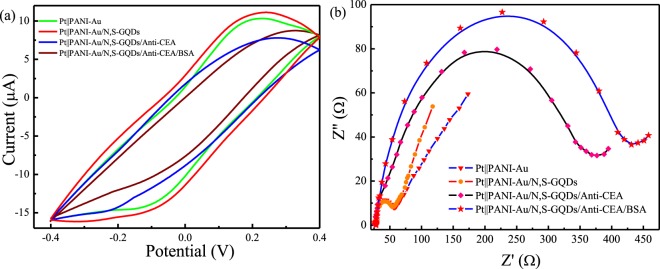
The (**a**) CV curves and (**b**) EIS spectra of Pt||PANI-Au based electrodes modified with N, S-GQDs and various biomolecules.

The electrochemical performance of step-wise immobilization procedure of the proposed immunosensor was further examined by CV and EIS. The CV curve was first used to probe the interface property of surface-modified electrodes at a scan rate of 100 mV s^−1^. As shown in Fig. 4a, an increase in current intensity is observed when the electrode surface is covered with N, S-GQDs@Au-PANI in comparison with pure Au-PANI. The redox couple at +0.21/−0.15 V is observed due to the electroactivity of PANI chain. However, the current intensity decreases gradually when biomolecules including anti-CEA antibody, BSA and CEA are added onto the surface of electrode, which attributed to the fact that the attachment of biomolecules inhibits the electron transfer process.

EIS is a well-established technique to characterize the feature of surface-modified electrodes and can be employed to elucidate the interfacial property as well as the change in electron transfer resistance (R_ct_). Figure 4b shows the EIS spectra of N, S-GQDs@Au-PANI electrodes modified with various biomolecules. Usually the Nyquist plot consists of a semicircle in the high frequency region follows by a linear line at low frequency. The semicircle is associated with R_ct_, which is dominated by the electron transfer-limited process, while the linear portion corresponds to the diffusion-controlled reaction^29^. As shown in Fig. 3b, the R_ct_ of Pt||PANI-Au electrode decreases after the modification with N, S-GQDs, indicating that N, S-GQDs are an excellent conducting material to accelerate the electron transfer. A dramatic increase in R_ct_ value of Pt||PANI-Au/N,S-GQDs/anti-CEA is observed in comparison with Pt||PANI-Au/N,S-GQDs. Similarly, BSA, being a large protein molecule, hinders the electron transfer, and results in the increase in diameter of semicircles successively. These results clearly indicate the successful immobilization of N, S-GQDs as well as biomolecules onto the surface of Pt||PANI-Au electrode. To confirm the importance of N, S-GQDs, we have also fabricated the immunosensor using Pt||PANI-Au/anti-CEA without the addition of N, S-GQDs. As shown in Fig. S5, the charge transfer resistance of Au-PANI and Au-PANI/anti-CEA is quite similar and the change in impedance is negligible in comparison with Au-PANI/N,S-GQDs/anti-CEA. This means that anti-CEA may not be attached onto the unmodified surface of Pt||PANI-Au electrode. The presence of N, S-GQDs in the composite helps the covalent linkage of Anti-CEA by the formation of amide bond between amine functional groups of Anti-CEA and carboxyl groups on N, S GQDs, resulting in the enhancement of analytical performance of electrode.

To understand the role of doped N and S elements in N, S-GQDs in improving the sensing performance, the sensing electrode was fabricated with the pristine GQDs. Figure S6 (Supplementary information) shows the EIS spectra of Pt||PANI-Au based electrodes modified with GQD, anti-CEA and N, S-GQDs/anti-CEA. The EIS spectrum of Pt||PANI-Au/GQDs/anti-CEA is almost identical to that of Pt||PANI-Au/GQDs except the shift in equivalent series resistance because of the added anit-CEA biomolecules. The R_ct_ values of Pt||PANI-Au/GQDs and Pt||PANI-Au/GQDs/anti-CEA are in the range of 34–36 Ω, which can be neglected in comparison with Pt||PANI-Au/N,S-GQDs/anti-CEA (350 Ω). It is also noted that the R_ct_ value of Pt||PANI-Au/GQDs is similar to that of Pt||PANI-Au (Fig. S5), clearly indicates that the pristine GQDs in the absence of N and S element is hard to attach onto the surface of Au-PANI because of the lack of Au-S thiol interaction, and subsequently decreases the immobilized amount of anti-CEA onto the electrode. To further elucidate the role of anti-CEA, the EIS spectra of Pt||PANI-Au/N,S-GQDs in the absence of anti-CEA was measured. It is clear that the change in R_ct_ of Pt||PANI-Au/N,S-GQDs after the addition of various concentrations of CEA is insignificant and does not follow any particular trend (Fig. S7, Supplementary Information). The small change in R_ct_ after the addition of CEA in the absence of anti-CEA is mainly attributed to the physical stacking of large CEA molecules onto the surface of Pt||PANI-Au/N,S-GQDs and, thus, hinders the electron mobility and pathway.

From the EIS and CV results, it is clear that the N, S-GQDs@Au-PANI can accelerate the electron transfer, while the anti-CEA antibody, BSA and CEA hinder the electron transfer to the electrode surface. Since the electron transfer resistance changes significantly during the addition of biomolecules, EIS can thus be utilized as an efficient analytical tool to develop the impedimetric immunosensor for the effective detection of CEA by Pt||PANI-Au@N, S-GQDs electrode.

### Analytical performance of the immunosensor

**Figure 5:**
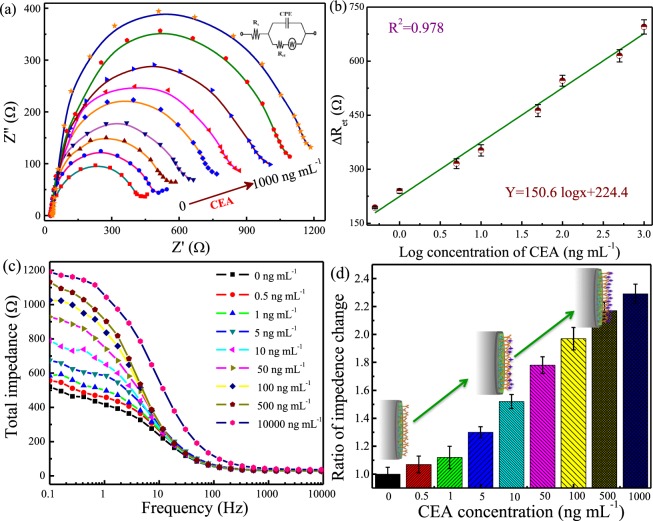
The (**a**) Nyquist plot of Pt||PANI-Au/N,S-GQDs/anti-CEA at various concentrations of CEA ranging from 0 to 1000 ng mL^−1^, (**b**) calibration curve of the immunosensor for the detection of CEA, (**c**) total impedance values with frequency before and after the exposure to various concentrations of CEA and (**d**) ratio of change in impedance with respect to the bare Pt||PANI-Au/N,S-GQDs/anti-CEA electrode without CEA at a frequency of 0.1 Hz.

By measuring the change in impedance of the electrochemical immunosensor, the EIS response of Pt||PANI-Au/N,S-GQDs/anti-CEA electrodes at various concentrations of CEA is shown in Fig. 5a. The detection of CEA is based on the degree of increased impedance of N, S-GQDs@Au-PANI electrode, which is attributed to the formation of CEA antigen-antibody bioconjugate on the electrode surface. The diameter of semicircle in Nyquist plot, which represents the charge transfer resistance at the electrode electrolyte interface (R_ct_), becomes large after the addition of CEA molecules, indicating the increase in resistance caused by the attachment of large-sized CEA onto the electrode surface. Moreover, the R_ct_ increases positively with the increase in CEA concentrations from 0.5 to 1000 ng mL^−1^. In addition, the equivalent circuit diagram used to fit the frequency range of 10 kHz–0.1 Hz is depicted in the inset of Fig. 5a, where *R*_s_ is the solution resistance, *CPE* is a constant phase element capacitance and *W* is the Warburg impedance. The fitted values of parameters at various CEA concentrations are shown in Table S1 (Supplementary Information). A significant change in R_ct_ is clearly observed after the addition of 0.5–1000 ng mL^−1^ CEA. The initial R_ct_ of 386 Ω is primarily contributed from the electron transfer between N, S-GQDs@Au-PANI and electrolyte. After the immobilization of CEA, large numbers of CEA cover on the surface of electrode and then occupy the bare region of Pt||PANI-Au/N,S-GQDs/anti-CEA electrode, resulting in the increase in R_ct_ from 580 Ω at 0.5 ng mL^−1^ to 1163 Ω at 1000 ng mL^−1^.

The value of Warburg impedance is in the range of 0.05–0.27 mΩ and has little influence on the impedance. The double layer capacitance usually decreases after immobilization of CEA, probably attributed to the increase in Debye length and the decrease in isoelectric point. Similar observation is also reported for various electrode–electrolyte systems^30,31^. It is noteworthy that the conjugation of CEA with anti-CEA onto the electrode surface may increase the solution resistance of charge transfer process, and, therefore, the R_s_ increases from 21.2 Ω in the absence of CEA to 31.2 Ω at 1000 ng mL^−1^ CEA. The value of n, an indicator of surface roughness, also increases slightly from 0.051 to 0.089 when the CEA concentration increases from 0 to 1000 ng mL^−1^ because of the formation of antigen-antibody bioconjugate. These results clearly indicate that the developed impedimetric immunosensor is sensitive to the added amounts of CEA concentration, which can possess a good analytical performance for the sensitive detection of CEA.

Figure 5b shows the calibration curve of CEA by Pt||PANI-Au/N,S-GQDs/anti-CEA using the EIS curve. The signal difference in R_ct_ values before and after the addition of CEA, denoted as R_et_, is adopted as the measurement index in the experiment. It is clear that the R_et_ increases proportionally upon increasing CEA concentration and a good linear relationship between R_et_ and logarithmic values of CEA concentration in the range of 0.5–1000 ng mL^−1^ is observed. The limit of detection (LOD), determined by the 3σ/S where σ is the standard deviation of the lowest signal and S is the slope of linear calibration plot, is 0.01 ng mL^−1^.

The impedimetric properties of the developed electrode at various concentrations of CEA is further examined by the total impedance (Z_tot_ = (Z_real_^2^ + Z_ima_^2^)^0.5^) with respect to the frequency range of 10 kHz–0.1 Hz. As presented in Fig. 5c, the total impedance in the low frequency region increases after the attachment of CEA, which indicates the increase in R_ct_. It is also clear that the R_ct_ in the low frequency region dominates the total impedance value, which has already been established in the Nyquist plots. Similarly, a well-defined calibration is also found in the range of 0.5–1000 ng mL^−1^ CEA. A comparative bar diagram of change in total impedance with increasing concentration of CEA is shown in Fig. 5d. The increase in impedance is found to be 2.28 fold with respect to the corresponding bare N, S-GQDs@Au-PANI electrode, which clearly represents the high specific nature of the anti-CEA conjugated N, S-GQDs@Au-PANI sensor towards CEA detection.

Table 1 summarizes the analytical performance of CEA by different electrochemical sensing probes. It is clear that most reported sensing probes exhibit a dynamic range of 2–3 orders of magnitude with LOD value of 0.01–5 ng mL^−1^. In this study, the N, S-GQDs@Au-PANI electrodes are highly sensitive toward CEA and the dynamic range can be up to 3–4 orders of magnitude with LOD of 0.01 ng mL^−1^, which is superior to the most of reported data shown in Table 1. The superior sensitivity of the developed impedimetric immunosensor is mainly attributed to the remarkable electro-conductivity of N, S-GQDs@Au-PANI, which can accelerate the electron transfer process between N, S-GQDs and electrode. The homogeneous and well-dispersed AuNP onto PANI nanowires also provides a large active sites for anchoring N, S-GQDs, and thereby allows anti-CEA to load onto N, S-GQDs@Au-PANI surface. In addition, the attachment of AuNP and N, S-GQDs through Au-thiol interaction is conducive to build a robust sensing platform with enhanced electrochemical response, leading to the high sensitivity and reliability that is required for EIS based sensor.

**Table 1:** Comparison of analytical performance of N, S-GQDs@Au-PANI electrode with other reported electrochemical based sensors.

Method	Sensing matrix^a^	Linear range (ng mL^−1^)	Detection limit (ng mL^−1^)	References
DPV	3D graphene foam	0.1–750	0.09	^36^
	IL-rGO-Au	0.01–100	0.01	^37^
	Au/rGO/PICA	0.02–90	0.02	^38^
CV	Graphene-MB-Au	5–60	5	^14^
	[Ag–Ag_2_O]/SiO_2_	0.5–160	0.14	^34^
	AuNP@nafion/FC@CHIT	0.01–150	0.03	^39^
EIS	Glutathione-Au	0.5–20	0.1	^32^
	Au/PPYGR	0.1–1000	0.06	^13^
	**N, S-GQDs@Au/PANI**	**0.5**–**1000**	**0.01**	**This work**

^a^IL: ionic liquid, rGO: reduced graphene oxide, PICA: poly (indole-6- carboxylic acid), MB: methylene blue, Ag_2_O: silver oxide, SiO_2_: silicon dioxide, CHIT: chitosan, and PPYGR: poly(ethylene glycol) pyrene butyric acid functionalized graphene.

### Selectivity stability and reproducibility of the immunosensor

**Figure 6:**
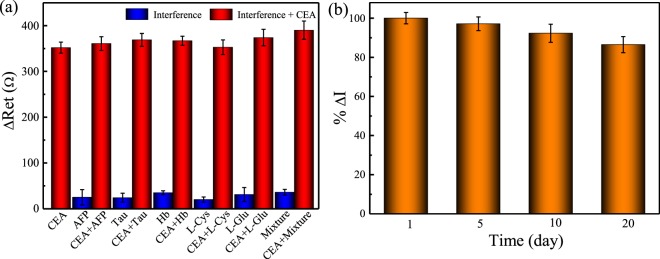
The (**a**) specificity and (**b**) stability of Pt||PANI-Au/N,S-GQDs/anti-CEA immunosensor.

The specificity of biosensor is an important factor for its real application to human serum. To characterize the selectivity of the proposed immunosensor, several proteins and cancer makers including alpha-fetoprotein (AFP), Tau protein (Tau), hemoglobin (Hb), L-cysteine (L-Cys) and L-glutamate (L-glu) at 10 ng mL^−1^ are used as potential interferences to evaluate the specificity through the comparison of the impedance response of 10 ng mL^−1^ CEA. Figure 6a shows the impedimetric response of Pt||PANI-Au/N,S-GQDs/anti-CEA immunosensor to the mixture of interference and CEA. The change in impedance (ΔR_et_) of Pt||PANI-Au/N,S-GQDs/anti-CEA is less than 40 Ω after the addition of pure interfering molecules. In contrast, the ΔR_et_ increases to 361–374 Ω when CEA is added to the individual interference. It is noteworthy that only 0.2–10% increase in ΔR_et_ in comparison with the pure CEA (in the absence of interference) is observed. Moreover, the ΔR_et_ increases from 350 ± 12 Ω for pure CEA to 390 ± 20 Ω for mixture, which corresponds to only 11.4% increase in response, after combining all the interfering molecules together with CEA, clearly indicating the superior specificity of Pt||PANI-Au/N,S-GQDs/anti-CEA immunosensor toward CEA detection.

The stability of the prepared immunosensor is also evaluated by keeping the electrodes at 4 °C and then the response of electrode was examined per 5 days. As shown in Fig. 6b, the impedimetric response of the immunosensor to CEA remains stable and the recovery of electrode is 98 ± 3% after 10 d of storage. Moreover, the impedimetric response can retain 92 ± 4% and 87 ± 4% of their original activity after 15 and 20 d of storage, respectively. This excellent long-term stability makes N, S-GQDs@Au-PANI nanocomposite an attractive material to serve as the biosensing probe for the electrochemically impedimetric immunosensor for stably long-term detection of CEA. Moreover, the reproducibility of the prepared immunosensor was evaluated by fabricating five different electrodes for the detection of 10 ng/mL of CEA. The relatively close impedimetric responses of the prepared electrodes are shown in Fig. S8 (Supplementary Information). The measured concentrations of CEA using five different electrodes are in the range of 10.4–11.5 ng mL^−1^ with an average of 10.9 ng mL^−1^, indicating the accuracy of the immunosensor is acceptable. In addition, the relative standard deviation (RSD) of the measurements is 6.8% suggesting the excellent reproducibility of immunosensor.

### Detection of CEA in human blood serum samples

As one of the tumor markers, the level of CEA in human serum and tissue is relatively low unless certain cancer cells are present. In order to validate the accuracy and feasibility of the proposed immunoassay for real sample analysis, standard addition method was used to detect the recovery of various concentrations of CEA in the serum samples. The impedimetric signal response of the immunosensor is recorded in 10× diluted serum samples to evaluate the precision of the developed immunosensor in real serum samples. Moreover, the CEA concentration at 5, 50 and 100 ng mL^−1^ is spiked to the serum samples to represent low, medium and high concentrations, respectively. As shown in Table 2, the recovery of 96.0–103.1% with relative standard deviation of 2.6–5.6% is observed in the CEA concentration range of 5–100 ng mL^−1^, suggesting that the proposed immunosensor is not affected by the biological interferences and can stably detect a wide concentration range of CEA in serum samples. It is noted that the sensing of CEA in real blood sample can provide more information on cancer diagnosis. However, the ingredient inside blood as well as anticoagulant added during the sampling procedure may severely interfere the direct measurement of CEA in blood samples. Further study is needed to develop an effective electrochemical sensing platform to detect cancer makers in blood.

**Table 2:** Recovery of various concentrations of CEA in human serum samples using N, S-GQDs@Au-PANI nanocomposite as the biosensing probe.

Serum Sample	Spiked (ng mL^−1^)	Detected (ng mL^−1^)	Recovery (%)	RSD (%) n = 5
Human serum sample	5	4.8	96.0	2.6
	50	47.9	95.8	5.6
	100	103.1	103.1	3.8

The spiked CEA concentrations are in the range of 5–100 ng mL^−1^.

## Discussion

In this study, a novel and ultrasensitive label-free impedimetric immunosensor has been successfully developed for the specifically detection of CEA using N, S-GQDs@Au-PANI as the biosensing platform. The AuNP with particle size of 4–11 nm are well-dispersed onto the surface of 30–50 nm PANI nanowires to provide large active area for N, S-GQD attachment. The uniform dispersion of AuNP onto PANI surface can increase the active sites of Au-PANI nanocomposites for effective decoration of N, S-GQDs by Au-thiol linkage. The Au-PANI increases the electron transfer process and provides a large active site for N, S-GQDs immobilization, while N, S-GQDs improve the electrochemical response and provide sufficient active sites to anchor anti-CEA. Therefore, the N, S-GQDs@Au-PANI nanocomposites can not only serve as the biosensing probe for antibody-antigen conjugates but also enhance the electrochemical performance on impedimetric detection of CEA. It is also noted that excess amounts of N, S-GQDs were added to allow the maximum attachment of N, S-GQDs onto the Au-PANI during the synthesis process and the attached amount of N, S-GQDs is highly dependent onto the Au loaded on the surface of Au-PANI. Although the N, S-GQDs content can be increased by the increase in Au amounts or by stacking of N, S-GQDs, the morphology as well as electrochemical performance of Au-PANI will be changed and then alter the electrochemical performance because of the increase in thickness and stacking effect of N, S-GQDs.

A dramatic increase in R_ct_ value of Pt||PANI-Au/N,S-GQDs/anti-CEA is observed in comparison with Pt||PANI-Au/N,S-GQDs. Since biomolecules is a poorly conductive material, the immobilization of anti-CEA antibody on the electrode would act as the electron-transfer blocking layer, and thereby hinders the access of electrons toward the electrode surface as well as increases the resistance. The obtained immunosensor exhibits superior detection ability toward CEA detection. A dynamic range of 4 orders of magnitude ranging from 0.5 to 1000 ng mL^−1^ with LOD value of 0.01 ng mL^−1^ is obtained. The highly sensitive Pt||PANI-Au/N,S-GQDs/anti-CEA immunosensor in comparison with the reported results^13,32^ is mainly attributed to the fact that N, S-GQDs have unique electro-optical property and the complex of N, S-GQDs@Au-PANI improved electron transfer capability. Moreover, the Au-PANI nanocomposite exhibits superior conductivity as well as serves as a support to provide a large surface area for the effective immobilization N, S-GQDs. These strategies make PANI-Au/N,S-GQDs promising electrode element for the effective development of immunosensors with high performances.

Several studies have developed the electrochemical sensors for the detection of CEA by conducting polymers and carbon dots in the presence of metal nanoparticles such as Au, Pt, and Pd^23,33,34^ have prepared a label-free immunosensor by the employment of Ag–Ag_2_O/SiO_2_ as the sensing probe for the determination of 0.5–160 ng mL^−1^ CEA. Li *et al*. have developed the water-dispersible graphene/amphiphilic pyrene derivative nanocomposite with high AuNP loading for CEA detection^13^. A linear relationship of 0.01–1000 ng mL^−1^ with LOD of 0.06 ng mL^−1^ was observed. Moreover, an enzyme-free biosensor by coupling cyclodextrin functionalized AuNP was developed for the electrochemical determination of CEA^33^. It is noteworthy that the detection mechanism of these works is mainly based on the well-known H_2_O_2_ catalysis reaction, where the reduction of H_2_O_2_ is served as a signal amplification platform. In this study, we have successfully developed the H_2_O_2_ free N, S-GQDs@Au-PANI nanoprobes for the direct impedimetric detection of CEA. Furthermore, the nontoxic usage of N, S-GQDs@Au-PANI in the absence of H_2_O_2_ makes this new class of impedimetric probe applicable for the selective and sensitive detection of CEA in biological samples. It is noteworthy that the added concentration of interfering molecules are 10 times higher than that of target CEA molecules and only around 10% of matrix effect is observed. These results indicate that Pt||PANI-Au/N,S-GQDs/anti-CEA immunosensor possesses high sensitivity and selectivity, which can effectively detect CEA specifically from the mixture containing all possibly competitive interfering molecules. In addition, the N, S-GQDs@Au-PANI can be applied for analysis of CEA in spiked serum samples and the recovery is in the range of (96.0 ± 2.6)–(103.1 ± 3.8)%. Results obtained in this study clearly demonstrate the superiority of the N, S-GQDs@Au-PANI nanocomposites to serve as a simple and cost-effective probing platform, which can open an avenue to fabricate the promising label-free impedimetric immunosensor for ultrasensitive and highly selective detection of cancer markers in clinical diagnosis and biomedical applications.

## Materials and Methods

### Chemicals

Citric acid, urea, gold(III) chloride trihydrate (HAuCl_4_·3H_2_O), toluene, aniline, sodium dihydrogen phosphate, disodium hydrogen phosphate, thiourea, N-(3-(dimethylamino)-propyl)-N′-ethylcarbodiimide hydrochloride (EDC), CEA and CEA antibody (anti-CEA) were purchased from Sigma-Aldrich. N-hydroxysuccinimide (NHS) was obtained from Alfa Aesar. Analytical grade of toluene and other reagents were used as received without further purification. All the solutions were prepared by using Milli-Q deionized water (18.2 MΩ cm) unless otherwise mentioned.

### Preparation of Au-PANI nanocomposites

Au-PANI nanocomposites were synthesized by the interfacially (water/toluene) oxidative polymerization of aniline in the presence of Au^3+^ ions. In a typical synthesis, aniline was first dissolved in 50 mL of toluene and then added into a round bottom flask containing 50 mL of 5 mM HAuCl_4_ aqueous solution to form a two-phase solution (Fig. 1a). As the reaction proceeded, the color of the lower aqueous layer turned into deep green, indicating the formation of conductive PANI. At the same time, the formation of aniline oligomer resulted in a color change from colourless to orange in the upper toluene phase. The reaction was allowed to continue for an additional 10 min, and then a stably fine layer of Au-PANI nanocomposite at the interface was dispersed in aqueous phase. In order to collect the green nanocomposite powder, the water phase was separated from the organic phase, filtered and dried under vacuum at 60 °C for 6 h to obtain a dark powder of Au-PANI nanocomposite^35^.

### Preparation of N, S-GQDs

The N, S-GQDs were prepared by a hydrothermal method with minor modification of our previously reported study by increasing the weight ratio of thiourea:citric acid to 1:1^22^. In brief, 0.23 g citric acid and 0.23 g thiourea were dissolved into 5 mL of deionized water under stirring conditions to form a clear solution. The solution was then transferred into a 20-mL of Teflon lined stainless steel autoclave tube and heated up to 160 °C for 4 h (Fig. 1b). The obtained brown suspension was added into ethanol solution and centrifuged at 5000 rpm for 5 min. The final product was re-dispersed into deionized water, dialyzed using 1 kDa dialysis membrane for 24 h and then stored at 4 °C for further use.

### Preparation of N, S-GQDs decorated Au-PANI nanowires

An excess amount of as-prepared N, S-GQDs was mixed with Au-PANI and stirred for 24 h to ensure the attachment of N, S-GQDs onto Au-PANI using the Au-thiol interaction. In order to collect the nanocomposite, the solution was centrifuged to separate the unattached N, S-GQDs, washed with DI water and dried under vacuum at 60 °C for 6 h to obtain a dark powder of N, S-GQDs@Au-PANI nanocomposites.

### Characterization of N, S-GQDs@Au-PANI nanowires

The morphology of N, S-GQDs@Au-PANI was characterized using JEOL JEM-2010 high-resolution transmission electron microscopy (HRTEM) at 300 kV. X-ray photoelectron spectroscopy (XPS) was performed with an ESCA Ulvac-PHI 1600 photoelectron spectrometer from Physical Electronics using Al Kα radiation photon energy at 1486.6 ± 0.2 eV. Fourier transform infrared (FTIR) spectra were determined using a Horiba FT720 spectrophotometer. X-ray diffraction (XRD) patterns were recorded using a Bruker D8 X-ray diffractometer with Ni-filtered Cu Kα radiation (λ = 1.5406 Å), and the thermal property of N, S-GQDs@Au-PANI was examined with thermogravimetric analysis (TGA) using Mettler Toledo DSC/TGA 3+ star system in N_2_.

### Fabrication of the immunosensor

The schematic diagram of the stepwise self-assembly procedure of the proposed label-free immunosensor is shown in Fig. 1c. The platinum electrode (Pt) was carefully polished with 1.0 and 0.3 µm alumina powder in sequence prior to the fabrication of immunosensor, and then thoroughly rinsed with ultrapure deionized water between each polishing step. The polished Pt electrode was first coated with 6 μL of 3.5 mg mL^−1^ N, S-GQDs@Au-PANI nanocomposite and dried in air. After drying for 1 h, the resultant Pt||PANI-Au@N,S-GQDs electrode was incubated with 6 μL of 10 μg mL^−1^ anti-CEA antibody by the chemical bonding between –COOH of N, S-GQDs and available F_c_ amine groups of anti-CEA antibody using EDC/NHS reaction. After the stepwise washing with PBS buffer, 3 μL of 1 wt% BSA solution were used to block the nonspecific binding sites between antigen and electrode surface. After 1 h of incubation, the Pt||PANI-Au@N,S-GQDs/anti-CEA was washed with ultrapure deionized water and incubated with various concentrations of CEA from 0.1 to 1000 ng mL^−1^ for 1 h at room temperature. The Pt||PANI-Au@N,S-GQDs/anti-CEA/CEA electrode was then washed with ultrapure deionized water to remove unbound CEA molecules for impedimetric measurements. Ultimately, the prepared immunosensor was stored at 4 °C for further usage.

### Electrochemical measurements

The electrochemical performance of the Pt||PANI-Au@N,S-GQDs/anti-CEA based electrode was characterized by cyclic voltammetry (CV) and the response of immunosensor to CEA was carried out by the electrochemical impedance spectroscopy (EIS). The CV curves were recorded in the potential window from −0.4 to 0.4 V at a scan rate of 100 mV s^−1^ to obtain the electroactivity of immunosensor and the EIS Nyquist plots were recorded in the frequency range of 10 kHz–0.1 Hz in 0.1 M PBS solution at pH 6.5. Electrochemical measurements were recorded using a potentiostat/galvanostat from Autolab PGSTAT 302 N electrochemical test system (Metrohm Autolab BV) in a conventional three-electrode system consisting of the sensing probe as the working electrode, Ag/AgCl as the reference electrode, and the platinum wire electrode as the counter electrode.

### Analysis of human serum samples

To evaluate the feasibility of using N, S-GQDs@Au-PANI nanoprobe for real-time biomedical application, standard addition method was used to determine CEA in commercial human serum samples (Sigma-Aldrich). Diluted human serum solutions were spiked with various amounts of CEA to obtain the concentration of 5–100 ng mL^−1^. The CEA concentration in serum sample was measured by similar procedure mentioned above and the recovery was calculated to evaluate the accuracy of the developed immunosensors. All the study was approved by the ethics committee and the institute review board (IRB) of National Tsing Hua University (IRB No. 10802HM004). All experiments were performed in accordance with relevant guidelines and regulations.

## Supplementary information

Supplementary Infomration

## References

[1] Xing T-Y (2017). Specific detection of Carcinoembryonic antigen based on fluorescence quenching of hollow porous gold nanoshells with roughened surface. ACS Appl. Mater. Interfaces.

[2] Li N-L (2017). A novel sandwiched electrochemiluminescence immunosensor for the detection of carcinoembryonic antigen based on carbon quantum dots and signal amplification. Biosens. Bioelectron..

[3] Hoseini S, Cheung N (2017). Acute myeloid leukemia targets for bispecific antibodies. Blood Cancer J..

[4] Gu X, She Z, Ma T, Tian S, Kraatz H-B (2018). Electrochemical detection of carcinoembryonic antigen. Biosens. Bioelectron..

[5] Su S (2017). Facile synthesis of a MoS_2_–prussian blue nanocube nanohybrid-based electrochemical sensing platform for hydrogen peroxide and Carcinoembryonic antigen detection. ACS Appl. Mater. Interfaces.

[6] Lin Z, Lv S, Zhang K, Tang D (2017). Optical transformation of a CdTe quantum dot-based paper sensor for a visual fluorescence immunoassay induced by dissolved silver ions. J. Mater. Chem. B..

[7] Wang D, Li Y, Lin Z, Qiu B, Guo L (2015). Surface-enhanced electrochemiluminescence of Ru@SiO_2_ for ultrasensitive detection of carcinoembryonic antigen. Anal. chem..

[8] Wang Z, Zong S, Wu L, Zhu D, Cui Y (2017). SERS-activated platforms for immunoassay: probes, encoding methods, and applications. Chem. rev..

[9] Xiao L (2017). Colorimetric biosensor for detection of cancer biomarker by au nanoparticle-decorated Bi_2_Se_3_ nanosheets. ACS Appl. Mater. Interfaces.

[10] Huang J-Y (2018). A high-sensitivity electrochemical aptasensor of carcinoembryonic antigen based on graphene quantum dots-ionic liquid-nafion nanomatrix and DNAzyme-assisted signal amplification strategy. Biosens. Bioelectron..

[11] Barman S, Hossain M, Yoon H, Park JY (2018). Trimetallic Pd@ Au@ Pt nanocomposites platform on-COOH terminated reduced graphene oxide for highly sensitive CEA and PSA biomarkers detection. Biosens. Bioelectron..

[12] Gosselin D (2017). Screen-printed polyaniline-based electrodes for the real-time monitoring of loop-mediated isothermal amplification reactions. Anal. chem..

[13] Li Y (2017). Water-dispersible graphene/amphiphilic pyrene derivative nanocomposite: High AuNPs loading capacity for CEA electrochemical immunosensing. Sens. Actuators B: Chem..

[14] Jin B (2014). Multi-nanomaterial electrochemical biosensor based on label-free graphene for detecting cancer biomarkers. Biosens. Bioelectron..

[15] Kumar V (2015). Ultrasensitive gold nanostar–polyaniline composite for ammonia gas sensing. Langmuir.

[16] Omar FS (2017). Binary composite of polyaniline/copper cobaltite for high performance asymmetric supercapacitor application. Electrochim. Acta.

[17] Ganganboina AB, Dutta Chowdhury A, Doong RA (2017). N-doped graphene quantum dots decorated V_2_O_5_ nanosheet for fluorescence turn off-on detection of cysteine. ACS Appl. Mater. Interfaces.

[18] Xie R (2016). Graphene quantum dots as smart probes for biosensing. Anal. Methods.

[19] Ganganboina AB, Chowdhury AD, Doong RA (2017). Nano assembly of N-doped graphene quantum dots anchored Fe_3_O_4_/halloysite nanotubes for high performance supercapacitor. Electrochim. Acta.

[20] Chowdhury AD, Ganganboina AB, Tsai YC, Chiu HC, Doong RA (2018). Multifunctional GQDs-Concanavalin A@Fe_3_O_4_ nanocomposites for cancer cells detection and targeted drug delivery. Anal. Chim. Acta.

[21] Ganganboina AB, Doong RA (2018). Functionalized N-doped graphene quantum dots for electrochemical determination of cholesterol through host-guest inclusion. Microchim. Acta..

[22] Anh NTN, Chowdhury AD, Doong RA (2017). Highly sensitive and selective detection of mercury ions using N, S-codoped graphene quantum dots and its paper strip based sensing application in wastewater, Sens. Actuators B: Chem..

[23] Yang Y (2017). A novel label-free electrochemical immunosensor based on functionalized nitrogen-doped graphene quantum dots for carcinoembryonic antigen detection. Biosens. Bioelectron..

[24] Ganganboina AB, Dutta Chowdhury A, Doong RA (2017). New avenue for appendage of graphene quantum dots on halloysite nanotubes as anode materials for high performance supercapacitors. ACS Sustain. Chem. Eng..

[25] Zhang Y, Keller D, Rossell MD, Erni R (2017). Formation of Au nanoparticles in liquid cell transmission electron microscopy: from a systematic study to engineered nanostructures. ACS Chem. Mater..

[26] Bogdanović U (2015). Interfacial synthesis of gold–polyaniline nanocomposite and its electrocatalytic application. ACS Appl. Mater. Interfaces.

[27] Zhu J, Lu Q, Chen C, Hu J, Liu J (2017). One-step synthesis and self-assembly of a luminescent sponge-like network of gold nanoparticles with high absorption capacity. J. Mater. Chem. C.

[28] Tsai YS (2013). TGF‐β1 Conjugated to gold nanoparticles results in protein conformational changes and attenuates the biological function. Small.

[29] Tsai Y-C, Doong RA (2016). Hierarchically ordered mesoporous carbons and silver nanoparticles as asymmetric electrodes for highly efficient capacitive deionization. Desalination.

[30] Diakowski PM, Xiao Y, Petryk MW, Kraatz H-B (2010). Impedance based detection of chemical warfare agent mimics using ferrocene-lysine modified carbon nanotubes. Anal. chem..

[31] Gerard M, Chaubey A, Malhotra B (2002). Application of conducting polymers to biosensors. Biosens. Bioelectron..

[32] Tang H, Chen J, Nie L, Kuang Y, Yao S (2007). A label-free electrochemical immunoassay for carcinoembryonic antigen (CEA) based on gold nanoparticles (AuNPs) and nonconductive polymer film. Biosens. Bioelectron..

[33] Zheng X (2018). Ultrasensitive enzyme-free biosensor by coupling cyclodextrin functionalized Au nanoparticles and high performance au-paper electrode. ACS Appl. Mater. Interfaces.

[34] Yuan Y (2010). A novel label-free electrochemical immunosensor for carcinoembryonic antigen detection based on the [Ag–Ag_2_O]/SiO_2_ nanocomposite material as a redox probe. J. Electroanal. Chem..

[35] Gangopadhyay R, Chowdhury AD, De A (2012). Functionalized polyaniline nanowires for biosensing. Sens. Actuators B: Chem..

[36] Liu J (2015). Three-dimensional electrochemical immunosensor for sensitive detection of carcinoembryonic antigen based on monolithic and macroporous graphene foam. Biosens. Bioelectron..

[37] Liu N, Ma Z (2014). Au–ionic liquid functionalized reduced graphene oxide immunosensing platform for simultaneous electrochemical detection of multiple analytes. Biosens. Bioelectron..

[38] Zhao D, Wang Y, Nie G (2016). Electrochemical immunosensor for the carcinoembryonic antigen based on a nanocomposite consisting of reduced graphene oxide, gold nanoparticles and poly (indole-6-carboxylic acid). Microchimica Acta.

[39] Shi W, Ma Z (2011). A novel label-free amperometric immunosensor for carcinoembryonic antigen based on redox membrane. Biosens. Bioelectron..

