# Supplementary material for: Graphene Quantum Dots Decorated Gold-Polyaniline Nanowire for Impedimetric Detection of Carcinoembryonic Antigen
Source: Sci Rep. 2019 May 10;9:7214. doi: 10.1038/s41598-019-43740-3 (PMC6510894; doi:10.1038/s41598-019-43740-3)
Supplement: Supplementary file 1 — Supplementary Infomration [file 41598_2019_43740_MOESM1_ESM.docx]

**Supplementary Information**

Graphene Quantum Dots Decorated Gold-Polyaniline Nanowire for Impedimetric Detection of Carcinoembryonic Antigen

*Akhilesh Babu Ganganboina^1^ and Ruey-an Doong ^1, 2, *^*

1. Department of Biomedical Engineering and Environmental Sciences, National Tsing Hua University, 101, Section 2, Kuang Fu Road, Hsinchu, 30013, Taiwan

2. Institute of Environmental Engineering, National Chiao Tung University, 1001 University Road, Hsinchu, 30010, Taiwan

*Ruey-an Doong, E-mail address: radoong@mx.nthu.edu.tw, Phone number: +886-3-5726785. Fax number: +886-3-5718649_._

**Table S1.** Electrochemically fitted parameters of Pt||BSA/Anti-CEA/N,S-GQDs@Au-PANI electrode obtained from impedance analysis based upon the proposed equivalent circuit after incubating with different concentration of CEA.

| CEA concentration (ng mL^-1^) | R_s_ (Ω) | n | W(mΩ) | R_ct_ (Ω) | χ^2^ |
| --- | --- | --- | --- | --- | --- |
| 0 | 21.2 | 0.051 | 0.05 | 386 | 1.8 × 10^-5^ |
| 0.5 | 23.6 | 0.051 | 0.04 | 580 | 4.1 × 10^-5^ |
| 1 | 26.1 | 0.053 | 0.21 | 639 | 1.9 × 10^-4^ |
| 5 | 26.3 | 0.052 | 0.28 | 694 | 4.5 × 10^-5^ |
| 10 | 26.9 | 0.080 | 0.04 | 725 | 8.8 × 10^-5^ |
| 50 | 27.9 | 0.086 | 0.04 | 815 | 1.4 × 10^-5^ |
| 100 | 28.3 | 0.087 | 0.04 | 937 | 1.5 × 10^-3^ |
| 500 | 28.5 | 0.089 | 0.03 | 1001 | 1.6 × 10^-3^ |
| 1000 | 31. 2 | 0.089 | 0.01 | 1163 | 1.5 × 10^-3^ |

**Figure S1.** XRD patterns of Au-PANI nanowires

**Figure S2.** Thermogravimetric analysis (TGA) of Au-PANI nanowires and N, S-GQDs@Au-PANI nanocomposites

**Figure S3.** The FTIR spectra of N, S-GQDs.

**Figure S4.** XPS survey scan of N, S-GQDs, Au-PANI nanowires and N, S-GQDs@Au-PANI nanocomposites.

**Figure S5**. EIS spectra of Pt||PANI-Au based electrodes modified with anti-CEA and N, S-GQDs/anti-CEA.

**Figure S6.** EIS spectra of Pt||PANI-Au based electrodes modified with GQD, anti-CEA and N, S-GQDs/anti-CEA.

**Figure S7.** EIS Spectra of Pt||PANI-Au/N,S-GQDs at various concentrations of CEA ranging from 0 to 100 ng mL^-1^

**Figure S8.** Impedimetric change in response of different electrodes treated with 10 ng mL^-1^ of CEA.
